# Increasing Incidence in Relapsing-Remitting MS and High Rates among Young Women in Finland: A Thirty-Year Follow-Up

**DOI:** 10.1155/2014/186950

**Published:** 2014-11-09

**Authors:** Marja-Liisa Sumelahti, Markus H. A. Holmberg, Annukka Murtonen, Heini Huhtala, Irina Elovaara

**Affiliations:** ^1^School of Medicine, University of Tampere, Arvo 312, 33014 Tampere, Finland; ^2^Tampere University Hospital, P.O. Box 2000, 33521 Tampere, Finland; ^3^School of Health Sciences, University of Tampere, 33014 Tampere, Finland

## Abstract

*Object*. Gender and disease course specific incidences were studied in high- and medium-risk regions of MS in Finland.* Methods*. Age- and gender-specific incidences with 95% CIs were calculated in 10-year periods from 1981 to 2010. Poser diagnostic criteria were used and compared with the McDonald criteria from 2001 to 2010. Association between age and diagnostic delay over time was assessed by using the Kruskal-Wallis test.* Results*. 1419 (89%) RRMS and 198 (11%) PPMS cases were included. RRMS incidence increased with the female to male ratio (F/M) from 4,2/10^5^ (F/M 1.9) to 9,7 (2.3), while that of PPMS decreased from 1,2 (1.6) to 0,7 (1.2). The use of McDonald criteria did not change the conclusion. The decreasing diagnostic delay and age at diagnosis in RRMS were associated within the 10-year periods and contrasted those in PPMS. Increasing female risk in RRMS was observed in the high-risk region.* Conclusion*. Increasing RRMS incidence and high female ratios shown in each age group indicate gender-specific influences acting already from childhood. A more precise definition of the risk factors and their action in MS is needed to provide a better understanding of underlying pathological processes and a rationale for the development of new preventive and treatment strategies.

## 1. Introduction

Increasing incidence in MS concerns the rising female to male (F/M) ratio [1] and relapsing-remitting (RRMS) type of the disease [2]. It has become apparent that environmental factors play an active role, but little is known about the factors that carry the gender- and disease-course-specific effects. The accumulated evidence indicates that latitudinal, genetic, and local environmental factors interact to cause MS, and a recent study has confirmed that the latitudinal effects are becoming more relevant at polar and intermediate latitudes for the RR phenotype, particularly in women [2].

The clinical and pathological spectrum of multiple sclerosis is heterogeneous [3]. Different subtypes are believed to share the same genetic architecture [4], and several lines of evidence suggest that multiple sclerosis susceptibility genes, particularly HLA-DRB1^*^15, influence the phenotypic expression of the disease, although some of the evidence on this aspect of the disease is conflicting [5]. The majority of MS cases may be classified as relapsing-remitting MS (RRMS) from onset and, in about 10–30% of cases, as primary progressive MS (PPMS). Clinical differences indicate a younger age at onset and female preponderance (F/M 2.2) in RRMS versus low F/M ratios (about 1.3) and older age in PPMS [6]. A unifying concept today holds that multiple sclerosis can be considered as one disease with different phenotypes [7].

The clinical segregation of disease courses is highly relevant, as the current disease-modifying treatments (DMT) are effective mainly against relapsing MS [8]. Recent changes in diagnostic criteria for MS have addressed the need for reliable disease type identification in MS. However, several issues regarding PPMS recognition have remained [9], although the criteria proposed in 2001 [10] and strict utilization of the latest McDonald criteria from 2010 [11] presumably help in avoiding diagnostic errors. Recent observations have pointed at stable incidence rates with PPMS, whereas RRMS incidence rates are rising [12, 13].

Finland is a country in northern Europe between the latitudes 60 and 70° and belongs to the high-risk areas of MS, together with Nordic areas [2]. MS incidence has increased significantly from 1981 to 2010 also in Finland, especially in the high-risk regions [14]. The high-risk areas in Finland are located in western Finland, especially in Seinäjoki and Vaasa, where respective total incidences were 12.5 per 10^5^ person-years and 8.3/10^5^ in 2010. The MS risk was twofold in Seinäjoki (SIR1.9) and significantly higher in Vaasa (1.2) compared with neighboring Pirkanmaa (comparator, 1.0). The incidence in Pirkanmaa was 6.7/10^5^, which is regarded as a medium-risk rate in Finland.

In this study we aim to study the disease-course- and gender-specific incidence trends in the high-risk districts of Seinäjoki and Vaasa and the medium-risk district of Pirkanmaa, while also considering the effect of changing diagnostic methods and criteria. In terms of the recent global trends in MS, we assume that the increase in RRMS is also seen in our study cohort. Given that the study populations are genetically and socioeconomically stable, we hypothesize that the environmental effects are reflected analogically in incidences in the districts, which would reflect the action of the underlying pathomechanism and common epigenetic factors in risk groups.

## 2. Material and Methods

The National Institute for Health and Welfare and local ethical standards committee approved retrospective scrutinizing of identified patient records in the hospitals under the study. Residence of cases was updated by personalized identification number and by year of diagnosis at Statistics Finland (http://www.stat.fi/).

**Figure 1:**
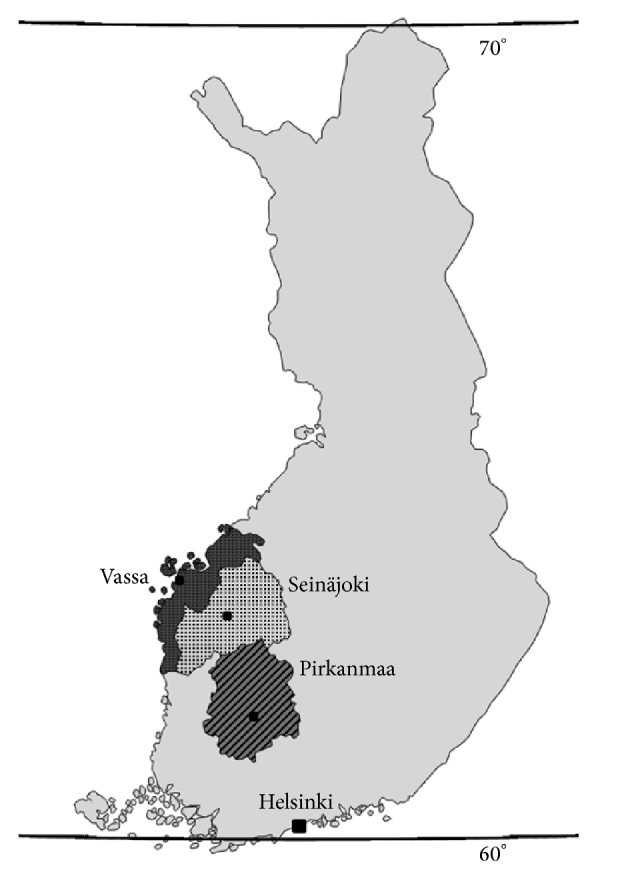
Map of Finland. Finland lies in Scandinavia, North Europe, between latitudes 60 and 70°N. Gulf of Bothnia in northern part of Baltic Sea limits the west coast. The total population was 5.4 million in 2010. Locations of Central Hospitals in Seinäjoki (dots) and Vaasa (dark grey) and University Hospital of Tampere at Pirkanmaa district (oblique lines) are pointed in the map.

A detailed description of study population, case collection, and case ascertainment is presented elsewhere [14]. Hospital districts under the study belong to Tampere University Hospital region in western Finland, shown in Figure 1. The high-risk area comprises the Seinäjoki and Vaasa Central Hospital districts and medium-risk area the Pirkanmaa district. The total population in the districts was 850,630 in 2010. Neurological services for diagnosis and treatment are evenly distributed among the hospitals. Magnetic resonance imaging (MRI) has been available from 1990 in Pirkanmaa and from 1993 in other hospitals.

Cases were included in the analysis when they fulfilled the criteria of clinically definite (CD) or laboratory-supported definite (LSD) MS by Poser et al. [15] between 1 January 1981 and 31 December 2010 and resided in the study districts in the year of diagnosis. Cases from 1 January 1981 to 31 December 2010 with diagnoses of morbus demyelinans and optic neuritis (340, 341 in ICD 8-9, G35, G37.4, and H46 in ICD 10) were first identified from the registries in the hospitals, where diagnosis was made by a neurologist. Patient documents were then scrutinized by the authors (MH, AM, and MLS). Case assessment included paraclinical tests and their time point, level, and results; for MRI, evoked potentials (EP) including visual, brainstem auditory, and sensory EPs; and cerebrospinal fluid (CSF). The year of onset and year of definite diagnosis were recorded, and information on specific onset symptoms was collected. The classification of disease course was reevaluated to ascertain whether the criteria of RRMS or PPMS were met [16]. To scrutinize the effect of changing diagnostic methods and criteria in the cohort, we reevaluated the cohort diagnosed from 2001 to 2010 by the McDonald criteria presented in 2001 [10].

The distribution of onset symptoms was calculated by disease course in the whole cohort. Initial MS symptoms were categorized in the following groups: motor (including pyramidal symptoms such as motor hemiparesis or paresis in the upper limbs and medullary symptoms, mainly motor paraparesis), brainstem (diplopia, trigeminal sensory symptoms, or facial dysfunction), visual (disturbances seen in optic or retrobulbar neuritis), sensory, and other symptoms (cerebellar dysfunction, fatigue or psychiatric problems, and epileptic seizures).

Age-adjusted gender- and disease-course-specific incidence rates per 10^5^ person-years were calculated with a 95% confidence interval (CI) from 1 January 1981 to 31 December 2010, and this period was divided into three subperiods: 1981–1990, 1991–2000, and 2001–2010. The incidence of the 5-year periods did not change our overall conclusions (data not shown). Furthermore, to avoid chance variation, we used 10-year periods in our calculations. Statistical analyses were performed using SPSS 9.0 for Windows. Correlation of diagnostic age (age at diagnosis) and delay (time from onset to diagnosis) across three time periods was conducted using Kruskal-Wallis tests [17] with Bonferroni adjustment for multiple comparisons. With the Kruskal-Wallis test, a chi-square statistic is used to evaluate the differences in mean ranks to assess the null hypothesis that the medians are equal across the groups. A *P* value of <0.05 with Bonferroni correction was considered statistically significant.

## 3. Results

From 1 January 1981 to 31 December 2010, a total of 1617 MS cases met the inclusion criteria in study districts (933 cases in Seinäjoki and Vaasa and 684 cases in Pirkanmaa). A total of 1105 (68%) women and 512 (32%) men were included. RRMS was observed in 1419 cases (89%) and PPMS in 198 cases (11%). F/M ratios in RRMS were 2.1 (965 women, 454 men) and in PPMS 1.2 (109/89).

Changes in age-adjusted incidences per 10^5^ person-years with 95% CIs for the three study periods 1981–1990, 1991–2000, and 2001–2010 are presented in Table 1. A twofold increase in RRMS (from 4.2 (3.7–4.6) to 9.7 (8.9–10.5)/10^5^) and a decrease in PPMS (from 1.2 (0.9–1.4) to 0.7 (0.5–0.7)) were observed. At the same time the female/male (F/M) ratios in RRMS increased from 1.9 to 2.3 and decreased in PPMS from 1.6 to 1.2.

**Figure 2:**
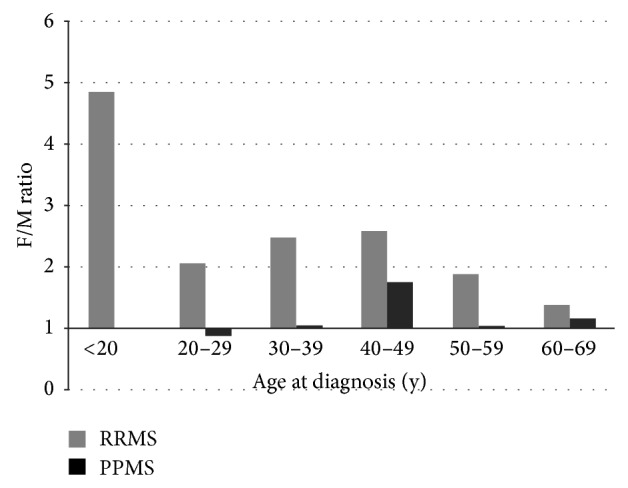
F/M ratios in ten-year age groups. F/M ratio in RRMS remained about twofold or higher from 20 to 59 years and in PPMS ratio was about 1 : 1, except for a slightly higher ratio in the 40–49 age group. The F/M ratio in RRMS is significant in age group younger than 20 years, where the ratio of women to men was 5 : 1 (*N* = 34/7 cases), and no PPMS cases were observed.

In RRMS group the mean age at diagnosis was 36.3 and in PPMS 45.3 years. We studied the age and gender effect by disease course by calculating the F/M ratios in each ten-year age group, presented in Figure 2 showing an exceptionally high F/M ratio of 5 : 1 (*n* = 34/7 cases) in RRMS age group <20 years. After this the distribution showed an approximately twofold ratio from 20 to 59 years in RRMS, while that in PPMS was 1 : 1.

**Figure 3:**
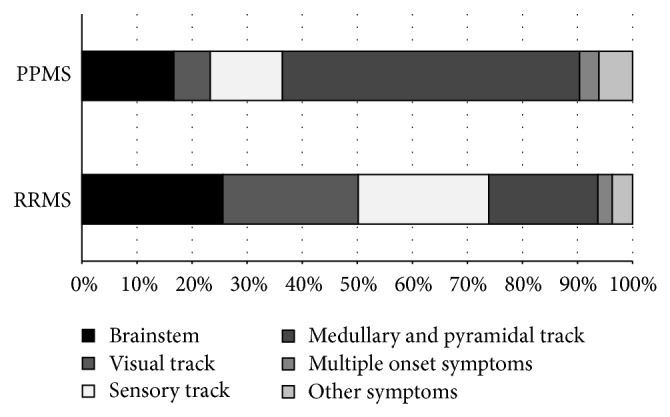
Distribution of onset symptoms in RRMS and PPMS groups diagnosed from 1981 to 2010.

In addition to age and gender difference, the distribution of onset symptoms showed a significant difference by disease course (*P* = 0.000), shown in Figure 3. In RRMS, brainstem (26%), visual (25%), and sensory symptoms (24%) were equally distributed and showed a female preponderance: F/M ratios were 2.0, 2.1, and 2.7. In PPMS, motor symptoms (total 53%) showed an F/M ratio of 0.8. (The distribution of onset symptoms showed no regional difference, not shown.)

**Figure 4:**
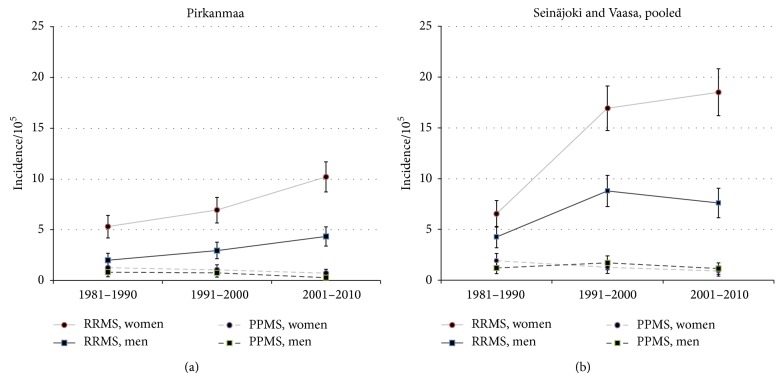
RRMS and PPMS incidence trends in medium-risk district Pirkanmaa (a) and high-risk districts Seinäjoki and Vaasa (b) from 1981 to 2010.

Incidences in both the medium-risk Pirkanmaa and western high-risk region showed increasing RRMS, decreasing PPMS, and parallel trends for men and women from 1981 to 2010 (Figures 4(a) and 4(b)). The increased incidence of RRMS showed regional differences: F/M ratios increased from 1.5, 1.9 to 2.3 in high-risk region, while ratios were stable (2.8, 2.3, and 2.3) in Pirkanmaa. This was true also in PPMS with the opposite downward incidence: F/M ratios declined (1.6, 0.7 to 0.8) in the high-risk district and increased (1.6, 1.4, and 2.6) at medium-risk district.

### 3.1. Case Ascertainment and Diagnostic Criteria

During the thirty years of follow-up both diagnostic criteria and case ascertainment have changed. Use of paraclinical tests in case ascertainment showed an increasing use of MRI scans: from 40% up to 97% in RRMS and from 20% to 93% in PPMS (Table 2). Scans including both brain and spinal cord remained low and pure spinal scans were rarely performed. The number of normal findings in the first diagnostic MRI scans was similar in the two groups (6.3% in RRMS and 8.6% in PPMS, not shown). There was no difference in MRI use between men and women (not shown).

Diagnostic cerebrospinal fluid (CSF) analysis has been used constantly, performed in 92% (*n* = 1299) of RRMS cases and 90% (*n* = 178) of cases with PPMS; no temporal change was seen (*P* = 0.4 in PPMS and 0.2 in RRMS). Positive findings (increased IgG index > 0.6 and oligoclonal bands in immunoelectrophoresis) were seen in 89% in RRMS and 91% in PPMS groups. The number of normal CSF findings increased in the RRMS group from 5.4% to 12%. Evoked potentials, including visual, brainstem auditory, and sensory potentials, were used decreasingly. In RRMS (*n* = 337, 24%), a positive result (prolonged latency) was seen in 76% (*n* = 256) of all studied EPs. In PPMS (*n* = 61, 31%), EP was positive in 97% (*n* = 49).

We have observed a decreasing diagnostic delay from onset symptoms to diagnosis in this cohort. The median diagnostic delay has decreased from 4.0 years to 2.0 years (chi-square test, *P* < 0.001) during the study periods [14]. This was mainly explained by improved diagnostics in MS. Decreasing delay was shown to concern especially RRMS course and male PPMS in this study (Table 3). The Kruskal-Wallis tests were used to compare the temporal relationship between the mean ages at diagnosis and diagnostic delays within the three ten-year periods from 1981 to 2010. Decreasing ages in RRMS were associated with decreasing diagnostic delays among both men (*P* = 0.035) and women (*P* = 0.036) over time. In PPMS group increasing ages for women associated with increasing delays (*P* < 0.0001), while increasing ages for men were observed in the presence of decreasing delays, result being nonsignificant.

To study the effect of changes in diagnostic criteria, we reevaluated cases diagnosed between 2001 and 2010 using the McDonald criteria published in 2001 [10] and calculated incidences for comparison with the group diagnosed with Poser criteria. Results are shown in Table 4. In McDonald group loss of 11 cases (24%) in PPMS and 57 cases (9%) in RRMS was seen, mainly due to normal or lacking MRI scans at the time point of first diagnosis (by Poser). However, the resulting 10-year incidence rates were similar using both sets of diagnostic criteria.

## 4. Discussion

Epidemiological observations in northern Europe [2] have pointed to a rising female and RRMS incidence, which was shown also here. During the thirty years of follow-up we also observed opposite incidence trends in RRMS and PPMS, characterized by differences in ages at diagnosis and distribution of gender and initial symptoms. The observation on decreasing age at diagnosis and diagnostic delay concerned both genders only in RRMS. In case of PPMS, there was an increase in age at diagnosis and a longer delay to diagnosis. Delay was higher among women with PPMS, which may be due to observed onset symptoms, which were more vague and unspecific compared to motor symptoms observed more often among men. However, together with the increasing use of MRI, up to 93% in PPMS and 97% in RRMS, and routine use of CSF in diagnostics, results indicate a more precise recognition of MS and reflect improved differential diagnostics, which are crucial in planning suitable treatment strategies for MS patients.

Explanations for the increasing MS incidence include increased awareness and improvements in case ascertainment and diagnostic accuracy [13]. In this cohort we were able to study the changes in case ascertainment during thirty years and compare incidence rates using both the Poser and McDonald criteria during the last decade 2001–2010 [10, 15]. A somewhat unexpected loss of 68 cases (4.2%) was seen using the McDonald criteria—57 in RRMS and 11 in PPMS—mainly due to normal or lacking MRI scans at the time point of diagnosis by the Poser criteria. However, eventual incidence rates were similar with the two sets of criteria. We thus believe that the clinical use of McDonald criteria, which underline the need of MRI use in MS diagnostics, confounded neither the temporal nor the regional comparisons and conclusions in this study.

Results during the last study decades 1991–2010 reflect the facilitation of access to MRI. The standardization of the performance and interpretation of MRI in the 2001 McDonald criteria have promoted an earlier diagnosis of RRMS in many cases of clinically isolated syndrome [18]. Nevertheless, criteria were criticized for rejecting historical accounts of symptoms and recommended the need for positive CSF for the diagnosis of PPMS. This may result in underdiagnosing of some cases and especially PPMS cases. The revision of criteria in 2005 has had a minor effect in our results.

However, the generally increasing integration of MRI into diagnostic work-ups seems to enhance the diagnosis among cases showing clinical relapses at onset. The need for early treatment [8] among these cases necessitates a reliable diagnosis, which appears in this study. Our observation that a low PPMS incidence was related to a somewhat less frequent use of MRI raises the question of a possible underestimation of PPMS cases during the follow-up. This is unlikely, as the number of PPMS cases was already very low in the beginning of the follow-up. Furthermore, the high rate of initial motor symptoms in PPMS, as was observed here, is associated with a relatively rapid disability progression leading to diagnosis [19–21]. The lengthy diagnostic delay and somewhat less frequent use of MRI, however, indicate that diagnosis is delayed in PPMS and it is diagnosed mainly on clinical grounds. Based on these data, we suggest early and repeated MRI in patients that present with symptoms consistent with MS in order to reach a disease-course-specific diagnosis and evaluate suitable therapy options.

The limitation in our study concerns the common classification problems in defining the clinical subtypes. Here, the disease course was categorized as either relapsing-remitting or primary progressive [16]. Recognition and diagnosis of PPMS using diagnostic criteria that are predisposed to inaccuracy, like the Poser criteria, have been criticized as leading to an underestimation of cases, whereas use of the McDonald criteria has been questioned due to validity and sensitivity issues [22, 23]. In general, the main issue in PPMS is the recall bias for initial symptoms, which can remain unidentified and lead to incorrect classification, regardless of the criteria used. Another caveat is the misclassification of secondary progressive cases as primary progressive, which is unlikely in this study cohort, considering the generally low number of PPMS cases and generally careful scrutiny of disease evolution in clinical practice. Explanations for the results in our former study [24] showing a tandem increase for the two progression types reflect the nowadays improved case recognition of PPMS, and, at the end of the day, this result contradicted neither the assumptions nor the conclusions in this study.

The strength of this study lies in a careful scrutiny of frequent medical assessments during the 30-year follow-up, which is believed to improve the reliability of the disease-course recognition. MS is diagnosed and treated by neurologists in public healthcare in Finland which is why we have a full coverage of MS patients in this population-based study. Furthermore, the neurological services and facilities are homogenously distributed throughout Finland, and the diagnostic work-up is convergent and based on International and National Current Care Guidelines [25].

It has been suggested that the differential gender distribution in MS reflects epigenetic factors and gene-environment interactions [26], including sex differences on the expression of candidate genes on the X or Y chromosome [27]. Given that the contributing genetic changes in populations are slow ones, the increasing female incidence in MS, rheumatoid arthritis in 1995–2007 [28], and other autoimmune diseases [29] indicates a crucial role of globally acting changes that affect autoimmune disease risk in the reproductive years of life. Environmental factors affecting women, such as contraception, diet, obesity, smoking, sunlight exposure, and vitamin D deficiency [27], are among the relevant lifestyle changes, as well as a higher age at first childbirth [30] and fewer pregnancies over a lifetime [31, 32]. These effects causing increased disease risk may be present already during the fetal period and in the childhood, supported by the fivefold F/M ratio among RRMS cases younger than 20 years as was seen in this study. This result corroborates observations of a higher relapse rate among females and the effect of age and disease duration on disease activity [33].

It is, however, unclear how environmental factors are shared between the two disease courses. To complicate inference, it is thought that the different phenotypes in MS are part of a disease spectrum modulated by individual genetic predisposition and environmental influences. In the case of RRMS, environmental factors that are able to cause functionally relevant changes in gene expression [34, 35] may be primarily associated with factors responsible for inflammatory reactions of a putative autoimmune nature in the central nervous system [36]. A better understanding of the factors that underlie the differential gender and disease-course distribution should shed more light on MS susceptibility factors overall.

Regional MS risk in Finland shows east-west gradient, and the high-risk region is located on the west coast. In this study we have shown that increasing incidence concerns exclusively RRMS cases in high-risk region Seinäjoki and Vaasa. Recent genome-wide scanning (GWAS) in MS has established the role of the HLA locus but also identified common variants associated with MS with low odds ratios [37]. To expose rare, high-impact alleles, a GWAS study was conducted in the high-risk internal isolate Seinäjoki district in Finland, where several large families with MS have been observed. Results showed a STAT3 gene association in MS [38], which implies a risk for another autoimmune disease in this cohort and has also raised the hypothesis that some genetic variants may be either more easily identified or etiologically more relevant in certain isolated populations. Also, family studies of MS have shown an increased risk of type 1 diabetes mellitus (DM), which also implies that certain genetic variants may increase susceptibility to autoimmune disease in general [39]. Childhood type 1 DM incidence follows MS incidence temporally [40] and may signal an increased penetrance of disease susceptibility genes in autoimmune diseases that may be more easily identified in genetic isolates or in families in high-risk regions of MS.

Increasing rates of relapsing MS in both medium- and high-risk districts indicate that a more precise definition of the genetic and environmental risk factors and their action in MS is needed to provide a better understanding of the underlying pathological processes and eventually to aid the development of preventive and treatment strategies. We conclude that initiatives to improve the use of population-based registers with linkages to independent national databases are needed to generate large international cohorts with shared demographic and clinical information [41].

## Figures and Tables

**Table 1:** Age-adjusted incidence of RRMS and PPMS per 10^5^ person-years with 95% CIs and F/M ratios from 1981 to 2010 in ten-year periods.

Disease course		RRMS				PPMS			
Period	Person-years	Number of new cases	Incidence		F/M	Number of new cases	Incidence		F/M
			I	95% CI			I	95% CI	
1981–1990	6208067	280	4.2	3.7–4.6	1.9	80	1.2	0.9–1.4	1.6
1991–2000	6142661	526	8.5	7.8–9.2	2.0	72	1.2	0.9–1.4	1.9
2001–2010	6326284	613	9.7	8.9–10.5	2.3	46	0.7	0.5–0.7	1.2

**Table 2:** Use of MRI scans in the diagnostics of MS by disease courses (RRMS and PPMS) in the study cohort diagnosed from 1981 to 2010.

Period	1981–1990		1991–2000		2001–2010		Total 1981–2010	
	*N*	%	*N*	%	*N*	%	*N*	%
RRMS cases	280		526		613		1419	
All scans/RRMS	113	40	446	85	599	97	1158	82
Brain	102	36	388	74	467	76	957	67
Spinal	5	2	13	2	20	3	38	3
Brain + spinal	6	2	45	9	112	18	163	12

PPMS cases	80		72		46		198	
All scans/PPMS	16	20	56	78	43	93	115	58
Brain	9	11	33	46	30	65	72	36
Spinal	1	1	7	10	2	4	10	5
Brain + spinal	6	8	16	22	11	24	33	17

**Table 3:** Mean age at diagnosis and diagnostic delay (years) presented by disease course and gender during the study periods 1981–1990, 1991–2000, and 2001–2010.

Disease course								
Period	RRMS				PPMS			
	Female		Male		Female		Male	
	Mean delay (y)	Mean age (y)	Mean delay (y)	Mean age (y)	Mean delay (y)	Mean age (y)	Mean delay (y)	Mean age (y)
1981–1990	4	36	3	37	2	44	4	43
1991–2000	2	37	2	36	3	44	3.5	43
2001–2010	2	35	1	35	3	53	2.5	50

**Table 4:** RRMS and PPMS incidences (I) and total incidence per 10^5^ person-years with 95% confidence intervals (CIs) by Poser and McDonald criteria in the whole study cohort from 2001 to 2010.

Criteria	Poser				McDonald			
	*N*	%	Incidence	95% CI	*N*	%	Incidence	95% CI
Disease course								
RRMS	613	93	9.7	8.9–10.5	556	94	8.8	8.1–9.5
PPMS	46	7	0.7	0.5–0.9	35	6	0.6	0.4–0.8

Total	659	100	10.4	9.6–11.2	591	100	9.3	8.5–10.1
